# Forecast of Malignant Peritoneal Mesothelioma Mortality in Italy up to 2040

**DOI:** 10.3390/ijerph18010160

**Published:** 2020-12-28

**Authors:** Enrico Oddone, Jordy Bollon, Consuelo Rubina Nava, Giada Minelli, Marcello Imbriani, Dario Consonni, Alessandro Marinaccio, Corrado Magnani, Francesco Barone-Adesi

**Affiliations:** 1 Department of Public Health, Experimental and Forensic Medicine, University of Pavia, 27100 Pavia, Italy; marcello.imbriani@unipv.it; 2 Occupational Medicine Unit (UOOML), ICS Maugeri IRCCS, 27100 Pavia, Italy; 3 Department of Translational Medicine, University of Eastern Piedmont, 28100 Novara, Italy; jbollon94@gmail.com (J.B.); corrado.magnani@med.uniupo.it (C.M.); francesco.baroneadesi@uniupo.it (F.B.-A.); 4 Department of Economics and Statistics Cognetti de Martiis, University of Turin, 10124 Turin, Italy; consuelorubina.nava@unito.it; 5 Statistics Service, Italian National Institute of Health (ISS), 00161 Rome, Italy; giada.minelli@iss.it; 6 Epidemiology Unit, Fondazione IRCCS Ca’ Granda Ospedale Maggiore Policlinico, 20122 Milan, Italy; dario.consonni@unimi.it; 7 Occupational and Environmental Medicine, Epidemiology and Hygiene Department, Italian Workers’ Compensation Authority (INAIL), 00187 Rome, Italy; a.marinaccio@inail.it

**Keywords:** peritoneal mesothelioma, asbestos, APC model, forecasts, public health, occupational medicine

## Abstract

Despite their differences, pleural and peritoneal mesothelioma are frequently lumped together to describe epidemic curves and to forecast future mesothelioma trends. This study aims to describe the malignant peritoneal mesothelioma (MPeM) epidemic in Italy (1996–2016) and to forecast future trends up to 2040 in order to contribute to the assessment of MPeM future burden. All MPeM deaths in Italy from 1996–2016 were collected (as provided by the Italian National Statistical Institute (ISTAT)) in order to estimate MPeM mortality rates for each 3-year period from 1996 to 2016. Poisson age-period-cohort (APC) models were then used to forecast MPeM future trends. Between 2017 and 2040, 1333 MPeM deaths are expected. The number of MPeM deaths, as well as mortality rates, are expected to constantly decrease throughout the considered period. Based on considering the information from this study, it can be concluded that the MPeM epidemic has probably already reached its peak in Italy.

## 1. Introduction

Peritoneum is the second most frequent site of malignant mesothelioma following the pleura. Malignant peritoneal mesothelioma (MPeM) has some peculiar features differentiating it from the pleural form [1], such as a lower male to female (M:F) ratio and a lower mean age at death (66 years) [2]. MPeM age-standardized incidence rates vary widely across different countries, generally ranging from 0.5 to about 3 cases per million population in men [3]. Worldwide, 4.5% of mesothelioma deaths in the period 1994–2008 were due to MPeM, with an age-adjusted mortality rate of 0.3 per million and a M:F ratio of 1.6:1, while the same ratio in pleural mesothelioma was 3.7:1 [2].

According to the Italian National Mesothelioma Register (ReNaM), the incidence of MPeM in Italy in 2014 was 1.7 per 1,000,000 in men and 1.0 in women [4].

Despite the clinical and epidemiological differences, pleural and peritoneal mesothelioma are often either lumped together to describe epidemic curves and to forecast overall mesothelioma trends, or MPeM cases are dropped and estimations are carried out based only on pleural mesothelioma data. Both approaches translate into a lack of information on MPeM epidemiology.

The aim of this paper is to describe MPeM mortality in Italy within the period 1996–2016 and to forecast future trends of this disease up to 2040 in order to contribute to the estimation of MPeM future burden.

## 2. Material and Methods

### 2.1. Data Source

Registrations of all MPeM deaths in Italy in 1996–2016 were collected from the Italian National Statistical Institute (ISTAT). A specific death code for MPeM (C45.1) was not available in Italy until 2003, when the tenth revision of the International Classification of Diseases was implemented. Thus, identification of cases for the period 1996–2003 was based on the individual assessment of death certificates, taking advantage of a study on MPeM previously conducted in the Italian National Multiple Causes of Death database of ISTAT [5].

Actual (1996–2016) and predicted (2017–2040) population data, stratified by year, gender, and age, were downloaded from the website of the National Institute of Statistics (http://demo.istat.it/index_e.html).

The present analysis was restricted to cases aged between 45 and 86 years of age, as MPeM is extremely rare before 45 (mortality rates of less than 0.92 per 1,000,000 person-year (p-y) were observed in Italy, both in men and women) and diagnosis is less certain at older ages.

### 2.2. Statistical Analysis

We estimated the MPeM mortality rates of men and women for each three-year period from 1996 to 2016, and we used Poisson age-period-cohort (APC) models to forecast MPeM future trends. To reduce collinearity issues, we used the parametrization and the sequential approach proposed in Carstensen [6], where age, period, and cohort effects are modeled on a continuous scale through the parametric smooth functions (here, the natural cubic splines). 

We ran different models with a number of spline parameters varying from 2 to 5, and we selected the model with the minimum Akaike information criterion (AIC) (Table S1). The considered model is characterized by three, three, and five parameters respectively for age, period, and cohort effects. We also conducted a secondary analysis using classic (i.e., categorical) APC models on three-year periods. In both approaches, estimated gender-specific coefficients of age, period, and cohort were then applied to population data to forecast the future number of cases. 

Data management and statistical analyses were performed using the *apc* and *Epi* R package (the code is in the Appendix A).

## 3. Results

Between 1996 and 2016, 1417 cases of MPeM were observed, most of which were found in men (878 cases, 62.0%). In all the three-year periods considered, the number of MPeM deaths was always higher in males, with M:F ratios between 1.45 and 1.88 (mean value: 1.64).

Observed mortality rates among men were broadly constant (1.29–1.59 per 1,000,000 p-y). Observed mortality rates among women never attained as high of values as those found in males, with the highest value of 1.01 per 1,000,000 p-y in 1999–2001 and rates reducing thereafter (Table S2).

Mortality rates increased by age, reaching over 5 and 2 per 1,000,000 p-y after 60 years of age, respectively, in men and women. The highest mortality rates were observed within the age class of 75–77 years for men born between 1931 and 1935 (10.54 per 1,000,000 p-y) and within the age class of 72–74 years for women born between 1925 and 1929 (5.14 per 1,000,000 p-y) (Tables S3 and S4; Figure S1). 

**Figure 1:**
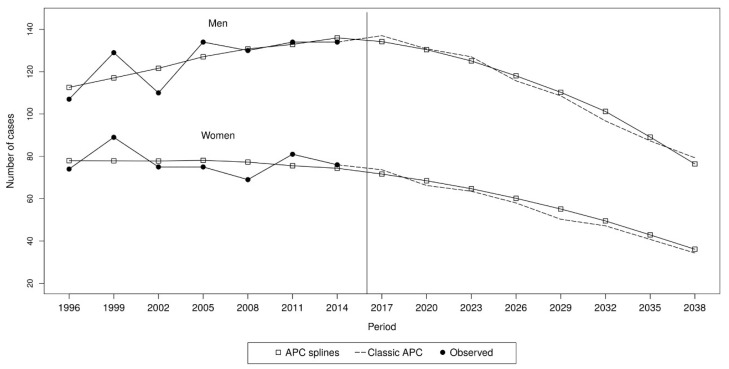
Observed and predicted number of deaths of malignant peritoneal mesothelioma under different age-period-cohort (APC) models, Italy, 1996–2040.

According to predictions of the APC model, in the period 2017–2040, 1333 MPeM deaths are expected, with 885 (66.4%) deaths in men and 448 (33.6%) deaths in women (Table S2). In both genders, the number of MPeM cases are predicted to constantly decrease throughout the considered period, reaching the lowest values in 2038–2040, which is expected to have approximately half of the maximum number of observed MPeM cases (Figure 1). The M:F ratio is predicted to remain quite constant (about 1.9–2.1:1). Mortality rates are predicted to decrease in a similar fashion as the absolute number of deaths, with lowest values at 0.86 and 0.40 cases per 1,000,000 p-y for men and women, respectively.

## 4. Discussion

Our study evaluated the evolution of the MPeM epidemic in Italy, providing both observed (1996–2016) and predicted mortality (2017–2040) figures.

A total of 1417 MPeM deaths were observed in Italy from 1996 to 2016, 992 of which occurred in the period 1996–2010. Considering that in the same period, 12,337 pleural mesotheliomas were observed [7], MPeM represented 7.4% of all mesothelioma deaths, a result that is higher than the worldwide observed percentage in a comparable time window [2]. Mean age at death (1996–2016) was 68.7 in men and 67.6 in women, and the male to female ratio was 1.63. These results are consistent with literature data.^2^

Over the considered period, the observed mortality rates ranged from 1.29 to 1.59 per 1,000,000 p-y and from 0.76 to 1.01 per 1,000,000 p-y for men and women, respectively. Considering that in Italy there is usually good agreement between incidence and mortality data for MPeM [5], our data suggest that Italy stands near the medium value of the ranges of MPeM incidence rates reported for the US and Europe (0.5–3 and 0.2–2 cases per million population, respectively, in men and women) [3].

Our results suggest that the number of MPeM cases is decreasing, with a peak of deaths already reached in 2014–2016 for men and in 1999–2001 for women. This outcome is different from predictions for pleural mesothelioma, whose peak has been predicted to occur sometime around 2020–2024 in Italy [7]. A potential misclassification between peritoneal mesothelioma and ovarian cancer is possible in women. Nonetheless, the similar trend observed in women and men, with the misclassification with ovarian cancer not possible in the latter group, suggests that no severe bias is tied with this potential issue. Several studies have found a clear association between asbestos exposure and subsequent MPeM onset [8,9,10], suggesting a potential role of higher asbestos exposures, especially in occupational settings [4,9,10]. Occupational asbestos exposures in Italy were more intense before 1980, levelling off and then later reducing [9,11]. Asbestos use was completely forbidden after 1992 by the enforcement of an Italian ban (Law 257/1992). Thus, the heaviest exposures in Italy could be related to the period 1960–1970, and this fact may explain the peak in MPeM cases that we observed around 2000–2010. These data could possibly explain differences between Italy and other countries, like Australia [12], in which MPeM has been predicted to increase, particularly in some age classes, in the near future.

## 5. Conclusions

Based on the results of this study, it can be concluded that the MPeM epidemic in Italy has probably already reached its peak, both in terms of the number of deaths and mortality rates. Nonetheless, 1333 MPeM deaths are expected in the period up until 2040. These figures are likely related to the high occupational asbestos exposure levels during the 1960s and the 1970s.

## Data Availability

The data used to describe mortality and forecast predictions were collected from the Italian National Statistical Institute (ISTAT).

## Supplementary Materials

The following are available online at https://www.mdpi.com/1660-4601/18/1/160/s1, Figure S1: Malignant peritoneal mesothelioma death rates per 1,000,000 persons-years by age and birth cohort. Italy, 1970–2014, Table S1: Comparisons of APC models by Akaike Information Criterion., Table S2: Number of observed and estimated malignant peritoneal mesothelioma deaths and corresponding death rates per 1,000,000 person-years. Italy,1996–2040., Table S3: Death rates (per 1,000,000 person-years) of malignant peritoneal mesothelioma in men by birth cohort and age at death. Italy, 1996–2016, Table S4: Death rates (per 1,000,000 person-years) of malignant peritoneal mesothelioma in women by birth cohort and age at death. Italy, 1996–2016.

## Appendix A. R Code

# Load library
    library(apc)
    library(Epi)
     
    # Load data frame with age, period, cases and person-years
    data = read.csv(data.csv)
     
    # Define a range of degree of freedom
    dof = 2:5
     
    # Create AIC vector
    aic_vec = c()
     
    # Create grid of degree of freedom
    par = as.matrix(expand.grid(rep(list(gdl.M), 3)))
     
    # Loop to search for best model in term of AIC
    for(i in 1:nrow(par)){
     # fit sequentially (parm = "AC-P") an apc splines (natural cubic splines: model = “ns”)
   	     model_fit = apc.fit(data, dist = "poisson", model = "ns", parm = "AC-P",
		       npar = as.vector(par[i, ]))
   	     aic_vec = c(aic_vec, model_fit$Model$aic)
    }
     
    # optimal model
 model_best = apc.fit(data, dist = "poisson", model = "ns", parm = "AC-P", npar = as.vector(par[which.min(aic_vec), ]))
		
