# Supplementary material for: Forecast of Malignant Peritoneal Mesothelioma Mortality in Italy up to 2040
Source: Int J Environ Res Public Health. 2020 Dec 28;18(1):160. doi: 10.3390/ijerph18010160 (PMC7796001; doi:10.3390/ijerph18010160)

**Figure S1**. Malignant peritoneal mesothelioma death rates per 1,000,000 persons-years by age and birth cohort. Italy, 1970–2014.


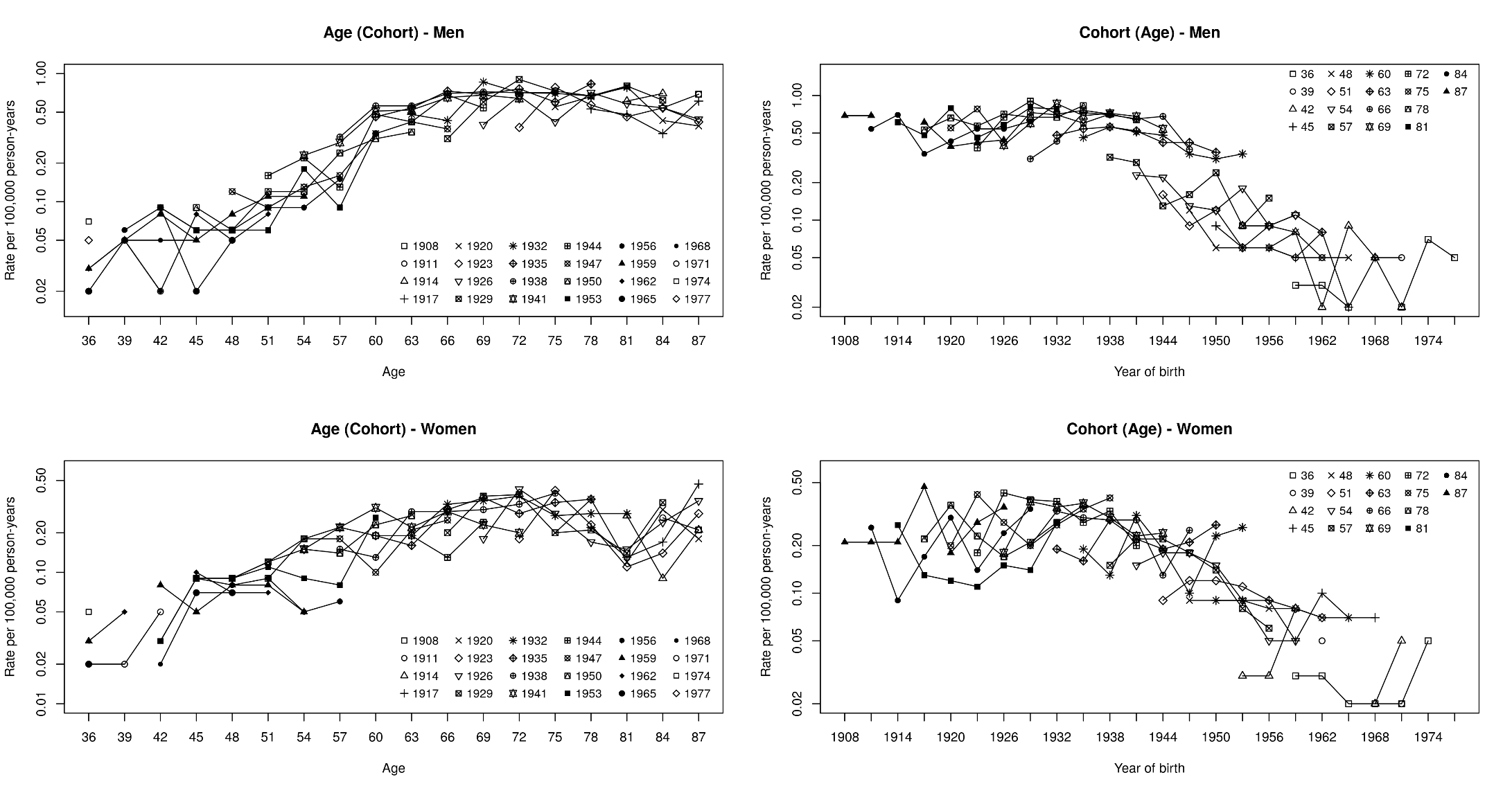

Supplement: Supplementary file 1 [file ijerph-18-00160-s001.zip › Figure S1.docx]
