# Supplementary material for: Forecast of Malignant Peritoneal Mesothelioma Mortality in Italy up to 2040
Source: Int J Environ Res Public Health. 2020 Dec 28;18(1):160. doi: 10.3390/ijerph18010160 (PMC7796001; doi:10.3390/ijerph18010160)
Supplement: Supplementary file 1 [file ijerph-18-00160-s001.zip › Table S1.docx]

**Table S1**. Comparisons of APC models by Akaike Information Criterion.

| **Model** | **Men** | **Women** |
| --- | --- | --- |
| Age-period-cohort  (Smoothed) | 478 | 441 |
| Age-period-cohort | 500 | 472 |
