# Supplementary material for: Forecast of Malignant Peritoneal Mesothelioma Mortality in Italy up to 2040
Source: Int J Environ Res Public Health. 2020 Dec 28;18(1):160. doi: 10.3390/ijerph18010160 (PMC7796001; doi:10.3390/ijerph18010160)
Supplement: Supplementary file 1 [file ijerph-18-00160-s001.zip › Table S2.docx]

**Table S2.** Number of observed and predicted malignant peritoneal mesothelioma deaths and corresponding death rates per 1,000,000 person-years. Italy, 1996-2040.

|  | **Male** | | | **Female** | | |
| --- | --- | --- | --- | --- | --- | --- |
| ***Periods*** | **Observed** | **Predicted** | **Mortality rate** | **Observed** | **Predicted** | **Mortality rate** |
| *1996-1998* | 107 | 113 | 1.29 | 74 | 78 | 0.84 |
| *1999-2001* | 129 | 117 | 1.56 | 89 | 78 | 1.01 |
| *2002-2004* | 110 | 122 | 1.32 | 75 | 78 | 0.85 |
| *2005-2007* | 134 | 127 | 1.59 | 75 | 78 | 0.84 |
| *2008-2010* | 130 | 131 | 1.52 | 69 | 77 | 0.76 |
| *2011-2013* | 134 | 133 | 1.55 | 81 | 76 | 0.88 |
| *2014-2016* | 134 | 136 | 1.52 | 76 | 74 | 0.81 |
| *2017-2019* | . | 134 | 1.52 | . | 72 | 0.77 |
| *2020-2022* | . | 130 | 1.47 | . | 68 | 0.74 |
| *2023-2025* | . | 125 | 1.40 | . | 65 | 0.70 |
| *2026-2028* | . | 118 | 1.32 | . | 60 | 0.65 |
| *2029-2031* | . | 110 | 1.24 | . | 55 | 0.60 |
| *2032-2034* | . | 101 | 1.14 | . | 49 | 0.54 |
| *2035-2037* | . | 89 | 1.00 | . | 43 | 0.47 |
| *2038-2040* | . | 76 | 0.86 | . | 36 | 0.40 |
