# Supplementary material for: Forecast of Malignant Peritoneal Mesothelioma Mortality in Italy up to 2040
Source: Int J Environ Res Public Health. 2020 Dec 28;18(1):160. doi: 10.3390/ijerph18010160 (PMC7796001; doi:10.3390/ijerph18010160)
Supplement: Supplementary file 1 [file ijerph-18-00160-s001.zip › Table S3.docx]

**Table S3** – Death rates (per 1,000,000 person-years) of malignant peritoneal mesothelioma in men by birth cohort and age at death. Italy, 1996-2016.

|  | **Periods** |  |  |  |  |  |  |  |  |  |  |  |  |  |  |  |  |  |  |  |
| --- | --- | --- | --- | --- | --- | --- | --- | --- | --- | --- | --- | --- | --- | --- | --- | --- | --- | --- | --- | --- |
|  | **1910-1914** | **1913-1917** | **1916-1920** | **1919-1923** | **1922-1926** | **1925-1929** | **1928-1932** | **1931-1935** | **1934-1938** | **1937-1941** | **1940-1944** | **1943-1947** | **1946-1950** | **1949-1953** | **1952-1956** | **1955-1959** | **1958-1962** | **1961-1965** | **1964-1968** | **1967-1971** |
| **Age (year)** |  |  |  |  |  |  |  |  |  |  |  |  |  |  |  |  |  |  |  |  |
| 45-47 |  |  |  |  |  |  |  |  |  |  |  |  |  | 0.59 | 0.61 | 0.58 | 0.54 | 1.24 | 0 | 0.45 |
| 48-50 |  |  |  |  |  |  |  |  |  |  |  |  | 0.86 | 0.59 | 0.31 | 0 | 0 | 0 | 0.68 |  |
| 51-53 |  |  |  |  |  |  |  |  |  |  |  | 1.01 | 0.87 | 0.90 | 0.61 | 0.87 | 1.09 | 1.23 |  |  |
| 54-56 |  |  |  |  |  |  |  |  |  |  | 1.95 | 4.11 | 2.36 | 1.21 | 2.47 | 0.88 | 0.54 |  |  |  |
| 57-59 |  |  |  |  |  |  |  |  |  | 1.92 | 2.34 | 1.75 | 2.09 | 1.53 | 0.94 | 2.34 |  |  |  |  |
| 60-62 |  |  |  |  |  |  |  |  | 5.97 | 5.29 | 4.46 | 4.28 | 2.73 | 3.46 | 2.83 |  |  |  |  |  |
| 63-65 |  |  |  |  |  |  |  | 4.78 | 5.49 | 4.11 | 8.12 | 4.03 | 2.51 | 1.27 |  |  |  |  |  |  |
| 66-68 |  |  |  |  |  |  | 4.31 | 5.45 | 7.29 | 7.12 | 5.49 | 6.10 | 4.47 |  |  |  |  |  |  |  |
| 69-71 |  |  |  |  |  | 5.73 | 7.63 | 4.16 | 5.28 | 6.36 | 7.34 | 7.50 |  |  |  |  |  |  |  |  |
| 72-74 |  |  |  |  | 6.59 | 4.92 | 5.12 | 7.68 | 7.43 | 8.04 | 6.51 |  |  |  |  |  |  |  |  |  |
| 75-77 |  |  |  | 6.92 | 5.85 | 4.46 | 7.29 | 10.54 | 9.18 | 7.80 |  |  |  |  |  |  |  |  |  |  |
| 78-80 |  |  | 4.85 | 10.00 | 6.98 | 5.19 | 5.42 | 6.94 | 7.58 |  |  |  |  |  |  |  |  |  |  |  |
| 81-83 |  | 3.32 | 4.69 | 4.28 | 7.06 | 6.42 | 7.38 | 10.30 |  |  |  |  |  |  |  |  |  |  |  |  |
| 84-86 | 4.78 | 7.77 | 8.66 | 5.88 | 2.40 | 9.72 | 4.76 |  |  |  |  |  |  |  |  |  |  |  |  |  |
