# Supplementary material for: Forecast of Malignant Peritoneal Mesothelioma Mortality in Italy up to 2040
Source: Int J Environ Res Public Health. 2020 Dec 28;18(1):160. doi: 10.3390/ijerph18010160 (PMC7796001; doi:10.3390/ijerph18010160)
Supplement: Supplementary file 1 [file ijerph-18-00160-s001.zip › Table S4.docx]

**Table S4**. Death rates (per 1,000,000 person-years) of malignant peritoneal mesothelioma in women by birth cohort and age at death. Italy, 1996-2016.

|  | **Period** |  |  |  |  |  |  |  |  |  |  |  |  |  |  |  |  |  |  |  |
| --- | --- | --- | --- | --- | --- | --- | --- | --- | --- | --- | --- | --- | --- | --- | --- | --- | --- | --- | --- | --- |
|  | **1910-1914** | **1913-1917** | **1916-1920** | **1919-1923** | **1922-1926** | **1925-1929** | **1928-1932** | **1931-1935** | **1934-1938** | **1937-1941** | **1940-1944** | **1943-1947** | **1946-1950** | **1949-1953** | **1952-1956** | **1955-1959** | **1958-1962** | **1961-1965** | **1964-1968** | **1967-1971** |
| **Age (year)** |  |  |  |  |  |  |  |  |  |  |  |  |  |  |  |  |  |  |  |  |
| 45-47 |  |  |  |  |  |  |  |  |  |  |  |  |  | 0 | 0.30 | 0.29 | 0.80 | 1.70 | 0.45 | 0.22 |
| 48-50 |  |  |  |  |  |  |  |  |  |  |  |  | 1.96 | 0.58 | 0.89 | 0.28 | 0 | 0.48 | 0 |  |
| 51-53 |  |  |  |  |  |  |  |  |  |  |  | 1.30 | 0.57 | 1.16 | 0.89 | 0.56 | 0.78 | 0.94 |  |  |
| 54-56 |  |  |  |  |  |  |  |  |  |  | 2.48 | 1.31 | 1.14 | 2.32 | 1.18 | 0.56 | 1.03 |  |  |  |
| 57-59 |  |  |  |  |  |  |  |  |  | 1.51 | 3.13 | 1.33 | 0.86 | 1.16 | 1.18 | 0.55 |  |  |  |  |
| 60-62 |  |  |  |  |  |  |  |  | 1.61 | 1.53 | 2.53 | 1.34 | 1.43 | 1.76 | 1.76 |  |  |  |  |  |
| 63-65 |  |  |  |  |  |  |  | 1.95 | 3.60 | 2.18 | 2.89 | 1.69 | 2.61 | 2.64 |  |  |  |  |  |  |
| 66-68 |  |  |  |  |  |  | 2.65 | 3.00 | 2.01 | 4.11 | 1.96 | 1.38 | 2.04 |  |  |  |  |  |  |  |
| 69-71 |  |  |  |  |  | 2.09 | 3.77 | 3.77 | 3.43 | 4.20 | 3.02 | 2.09 |  |  |  |  |  |  |  |  |
| 72-74 |  |  |  |  | 3.68 | 5.14 | 3.94 | 2.50 | 1.78 | 3.35 | 2.41 |  |  |  |  |  |  |  |  |  |
| 75-77 |  |  |  | 3.67 | 2.77 | 3.92 | 1.52 | 4.53 | 3.76 | 3.83 |  |  |  |  |  |  |  |  |  |  |
| 78-80 |  |  | 2.24 | 3.56 | 1.30 | 0.85 | 1.23 | 4.50 | 1.60 |  |  |  |  |  |  |  |  |  |  |  |
| 81-83 |  | 1.26 | 1.75 | 0.58 | 3.43 | 0.96 | 1.86 | 3.17 |  |  |  |  |  |  |  |  |  |  |  |  |
| 84-86 | 1.62 | 3.14 | 2.16 | 0.70 | 0.59 | 2.89 | 4.35 |  |  |  |  |  |  |  |  |  |  |  |  |  |
